# Erratum to: The Fixed-Dose Combination of Olmesartan/Amlodipine Was Superior in Central Aortic Blood Pressure Reduction Compared with Perindopril/Amlodipine: A Randomized, Double-Blind Trial in Patients with Hypertension

**DOI:** 10.1007/s12325-015-0186-4

**Published:** 2015-02-25

**Authors:** Luis Ruilope, Angie Schaefer

**Affiliations:** 1 Hypertension Unit, Hospital 12 de Octubre, 28041 Madrid, Spain; 2 Department of Preventive Medicine and Public Health, Universidad Autonoma, Madrid, Spain; 3 Daiichi Sankyo Europe GmbH, Zielstattstrasse 48, 81379 Munich, Germany

## Erratum to: Adv Ther (2013) 30(12):1086–1099 DOI: 10.1007/s12325-013-0076-6

Some errors have been noted in the above-mentioned paper subsequent to publication and so the following corrections should be highlighted.

On page 1091 under the heading ***Treatment Patterns***, the sentence “Overall, the mean duration of treatment was 152.6 days, and there were no major differences between the two treatment groups, including the duration of add-on HCTZ treatment” should read “Overall, the mean duration of treatment was 151.2 days, and there were no major differences between the two treatment groups, including the duration of add-on HCTZ treatment.” It should be clarified that 152.6 days was the mean duration of the double-blind treatment phase.

On page 1092 in ***Fig. 3***, the *p* value for the FAS group should read *p* = 0.0005, and not *p* < 0.0001.

On page 1095 under the heading ***Safety/Tolerability***, the sentence “The most common adverse event was peripheral edema, which was reported in 17.8% of OLM/AML patients and 18.1% of PER/AML recipients” should read “The most common adverse event was peripheral edema, which was reported in 18.4% of OLM/AML patients and 17.8% of PER/AML recipients.”

On page 1095 in ***Table 2***, the values in the row showing the frequencies of peripheral edema which are currently: 43 (17.8) in the OLM/AML column, 88 (18.1) in the PER/AML column, and 45 (18.4) in the total column; should read: 45 (18.4), 43 (17.8), and 88 (18.1), respectively.

